# Vedolizumab use in patients with inflammatory bowel diseases undergoing surgery: clinical trials and post-marketing experience

**DOI:** 10.1093/gastro/goz034

**Published:** 2019-08-21

**Authors:** Bo Shen, Aimee Blake, Karen Lasch, Michael Smyth, Fatima Bhayat

**Affiliations:** 1 Center for Inflammatory Bowel Disease, Digestive Disease and Surgery Institute, Cleveland Clinic, Cleveland, OH, USA; 2 Global Patient Safety Evaluation, Takeda Pharmaceuticals International Co., Cambridge, MA, USA; 3 US Medical Office, Takeda Pharmaceuticals USA Inc., Deerfield, IL, USA; 4 Global Medical Affairs, Takeda Development Centre Europe Ltd, London, UK; 5 Kyowa Kirin International plc, Chertsey, UK

**Keywords:** inflammatory bowel disease, colorectal surgery, vedolizumab

## Abstract

**Background:**

Patients with inflammatory bowel diseases frequently require surgery, but immunotherapies used in disease management may increase the risk of post-operative complications. We investigated frequencies of post-operative complications in patients who received vedolizumab—a gut-selective antibody approved for the treatment of moderately to severely active ulcerative colitis and Crohn’s disease—in clinical-trial and post-marketing settings.

**Methods:**

This post hoc analysis of safety data from GEMINI 1, GEMINI 2, and long-term safety studies included patients who had had colectomy or bowel surgery/resection. Data from the post-marketing Vedolizumab Global Safety Database were also analysed (data cutoff point: 19 May 2016). Adverse events relating to post-operative complications were identified using *Medical Dictionary for Regulatory Activities* preferred terms.

**Results:**

Of 58 total surgeries in patients included in GEMINI 1 and GEMINI 2, post-operative complications were reported for 3/51 vedolizumab-treated patients (5.9%) and 1/7 placebo-treated patients (14.3%). In the long-term safety study, 157/2,243 patients (7%) had colectomy or bowel surgery/resection; of these 157 patients who underwent surgery, 11 (7%) experienced a post-operative complication. Median time between last pre-operative vedolizumab dose and surgery was 23 days in GEMINI 1, 20 days in GEMINI 2, and 39‒40 days in the long-term safety study. In the post-marketing setting, based on data covering approximately 46,978 patient-years of vedolizumab exposure, post-operative complications were reported in 19 patients.

**Conclusions:**

In clinical trials, complications of colectomy and bowel surgery/resection appeared infrequent, with minimal difference between vedolizumab and placebo. The frequency of post-operative complications in the post-marketing setting appears low.

## Introduction

Patients with inflammatory bowel disease (IBD) may require surgical intervention if they show inadequate response to immunosuppressive therapies or develop colitis-associated cancer. Approximately 25%–30% of patients with ulcerative colitis (UC) and 70%–75% of patients with Crohn’s disease (CD) require surgery during the course of their disease [1]. Common procedures include restorative proctocolectomy with ileal pouch–anal anastomosis in patients with UC and bowel resection with anastomosis and stricturoplasty in patients with CD [2, 3]. In the post-operative period, suboptimal wound healing can result in complications, such as superficial or deep space surgical-site infections (SSIs), anastomotic leakage, or pelvic sepsis [4]. The risk of post-operative complications may be exacerbated by immunosuppressive therapies, such as corticosteroids, or biologics including tumor necrosis factor alpha (TNFα) antagonists [5, 6]. According to several systematic reviews and meta-analyses, pre-operative use of TNFα antagonists may modestly increase the risk of post-operative complications in patients with IBD [7–11]; however, current evidence is inconclusive [12, 13].

Vedolizumab (ENTYVIO^®^; Takeda Pharmaceuticals, Deerfield, IL, USA)—a humanized monoclonal antibody that specifically targets α_4_β_7_ integrin and prevents gut-selective lymphocyte trafficking—is approved for the treatment of adults with moderately to severely active UC and CD [14]. It is unknown whether blocking T-lymphocyte migration to the intestines affects normal post-operative wound healing or increases the risk of post-operative complications [4].

The frequency of post-operative complications in patients with IBD who received pre-operative or perioperative vedolizumab was investigated in several single-center, retrospective studies [4, 15–20]. Post-operative complications, including SSIs, were reported to occur in 13%‒53% of vedolizumab-treated patients within 1 month of surgery [4, 15–17]. Analyses by Lightner *et al*. [4, 18, 19], most of which used data from a single US electronic medical records database and one multicenter study [21], found that vedolizumab-treated patients had a significantly higher number of SSIs than TNFα antagonist-treated patients. These findings were robust to univariate and multivariate analyses adjusting for potential confounding factors [4, 19, 21]. However, other studies reported that perioperative use of vedolizumab was not associated with short-term (<30 days) post-operative complications in patients with IBD [15–17, 20] and that the risk was not greater than that reported for anti-TNFα or non-biologic therapy [15, 16, 20]. A recent meta-analysis concluded that pre-operative vedolizumab treatment in patients with IBD did not appear to be associated with an increased risk of post-operative infectious or overall post-operative complications compared with either pre-operative anti-TNFα therapy or non-biologic therapy [22].

Based on evidence from observational studies, the impact of vedolizumab on post-operative complications in patients with IBD is unknown. Conflicting evidence prompted us to investigate post-operative complications with vedolizumab using different data sources. Post-operative outcomes were assessed using data from prospective, phase 3 vedolizumab clinical trials (GEMINI 1 and GEMINI 2) [23, 24], as well as data collected in an ongoing long-term safety study (LTS) and from individual case safety reports from the post-marketing Vedolizumab Global Safety Database (GSD).

## Methods

### Data sources

#### GEMINI 1 and GEMINI 2 trials

The efficacy and safety of vedolizumab 300 mg were investigated in two phase 3, randomized, placebo-controlled trials in adults with moderately to severely active UC (GEMINI 1, NCT00783718) or CD (GEMINI 2, NCT00783692; Supplementary Figure 1). As described previously, all patients had a history of unsuccessful IBD-related therapy (corticosteroids, immunomodulators, or TNFα antagonists) and were excluded if they had undergone major abdominal surgery (Supplementary File 1) [23, 24].

In this analysis, the vedolizumab population comprised patients who responded to vedolizumab induction therapy (6 weeks) and were assigned vedolizumab maintenance therapy (dosing every 4 or 8 weeks), and patients with no clinical response at week 6 who were assigned vedolizumab (dosing every 4 weeks) for the maintenance phase. Patients were exposed to vedolizumab for a maximum of 52 weeks. The placebo population was assigned placebo during both induction and maintenance phases [25].

#### GEMINI LTS study

The GEMINI LTS study (NCT0079033) evaluated the safety and efficacy of vedolizumab 300 mg every 4 weeks in adult patients who had previously participated in vedolizumab clinical studies (rollover patients), as well as patients naive to vedolizumab treatment (*de novo* patients; Supplementary Figure 1 and Supplementary File 1) [26, 27]. Most patients in this study were recruited from the GEMINI 1 [23], GEMINI 2 [24], and GEMINI 3 [28] (NCT01224171, patients with CD) trials, as well as an open-label, phase 2 trial (NCT00619489) [29]. The total duration of vedolizumab treatment varied, with the maximum duration of exposure being 196 weeks.

#### Post-marketing safety data

All adverse-event reports of post-operative complications received by the license holder, Takeda Pharmaceutical Company Ltd, since the approval date of vedolizumab (20 May 2014) are held in the Vedolizumab GSD. Sources of these reports include spontaneous reports from patients, healthcare professionals, and regulatory authorities; solicited reports from patient-support programs and market-research programs; and reports extracted from the literature. This summary may include adverse events reported to Takeda that have previously been reported in separate publications.

### Outcomes analysed

#### Clinical trials

The incidences of IBD-related colectomies and other bowel surgeries/resections were calculated for each trial. Information on procedure type, such as one-stage or two-stage resection, was not collected because surgery was not an end-point of the clinical studies.

In GEMINI 1 and GEMINI 2, serious and non-serious adverse events were collected for up to 16 weeks after the final study dose of vedolizumab or until patients were enrolled into the GEMINI LTS study. In the GEMINI LTS study, non-serious adverse events were collected for up to 16 weeks after the final study dose of vedolizumab. Serious adverse events that occurred at any time after study completion and considered to be related to vedolizumab were reported. Adverse events relating to post-operative complications and to deaths that occurred <30 days after surgery were identified using the *Medical Dictionary of Regulatory Activities* (MedDRA) high-level term group *sepsis, bacteremia, viremia and fungemia not elsewhere classified* and a selection of the MedDRA-preferred terms representative of the medical concept of post-operative wound healing (Supplementary File 2). Adverse event and serious adverse event were defined in line with the US Food and Drug Administration Code of Federal Regulations (Title 21, Volume 5) [30].

#### Post-marketing safety reports

All adverse-event reports of post-operative complications received between the approval date of vedolizumab and the data cutoff point (19 May 2016) were identified in the Vedolizumab GSD using MedDRA search terms (Supplementary File 2). Information on the type of adverse events after surgery and death was reported. Adverse events were categorized according to serious and non-serious events, type of IBD, and prior TNFα antagonist exposure.

### Statistical analysis

Continuous variables were expressed as mean ± standard deviation (SD) and median (range) values, and categorical variables as *n* (%). Differences in values between treatment groups were not assessed using statistical tests because there was insufficient statistical power to permit meaningful comparisons.

### Ethical approval

The GEMINI clinical trials and LTS study reported here have been published elsewhere and were conducted in accordance with the ethical principles in the Declaration of Helsinki; all appropriate study documentation was reviewed by the institutional review board and independent ethics committee according to local regulations. Our research also included data from the post-marketing setting; this did not involve any patient investigations that necessitated ethical approval at the national or institutional level.

## Results

### IBD-related surgery in the clinical trials

In GEMINI 1 and GEMINI 2, IBD-related colectomies and other bowel surgeries/resections were reported in 51 patients (3.6%) receiving vedolizumab and 7 patients (2.4%) receiving placebo. Baseline demographics and disease characteristics of 58 patients who underwent IBD-related surgery in the GEMINI 1 and 2 trials are provided in Table 1 (baseline characteristics for all patients in the clinical trials are provided in Supplementary Table 1). The mean exposure time was 258.5 ± 118.0 days for vedolizumab and 181.4 ± 118.4 days for placebo in GEMINI 1, and 246.8 ± 112.4 days for vedolizumab and 203.0 ± 127.0 days for placebo in GEMINI 2. The median number of days between the last dose of treatment and surgery was 23 (8‒88) for vedolizumab and 8 (6‒23) for placebo in GEMINI 1, compared with 20 (1‒92) and 10 (6‒18) in GEMINI 2 for vedolizumab and placebo, respectively.

**Table 1.** Baseline demographics and disease characteristics of patients who underwent IBD-related surgery during the clinical studies

Parameter	GEMINI 1		GEMINI 2		GEMINI LTS study	
	Vedolizumab (*n* = 15)	Placebo (*n* = 3)	Vedolizumab (*n* = 36)	Placebo (*n* = 4)	UC (*n* = 55)	CD (*n* = 102)
Baseline						
Age, years	37.0 ± 8.6	39.8 ± 12.3	34.4 ± 11.7	34.1 ± 9.8	40.6 ± 16.2	36.6 ± 12.4
Female	7 (46.7)	1 (33.3)	16 (44.4)	3 (75.0)	29 (52.7)	60 (58.8)
Caucasian	13 (86.7)	2 (66.7)	35 (97.2)	4 (100.0)	51 (92.7)	97 (95.1)
Body weight, kg	67.0 ± 15.5	81.4 ± 17.3	71.1± 24.5	64.5 ± 23.0	72.7 ± 17.9	69.0 ± 15.1
Current smoker	0 (0.0)	0 (0.0)	5 (13.9)	1 (25.0)	1 (1.8)	30 (29.4)
Former smoker	3 (20.0)	2 (66.7)	9 (25.0)	1 (25.0)	20 (36.4)	23 (22.5)
Duration of disease, years	5.5 ± 5.6	3.0 ± 1.7	9.0 ± 6.0	7.4 ± 6.2	6.7 ± 5.9	11.8 ± 9.5
Disease duration ≥7 years	5 (33.3)	0 (0.0)	19 (52.8)	2 (50.0)	19 (34.5)	64 (62.7)
Baseline disease activity (Mayo score)	9.2 ± 1.6	9.7 ± 2.3	NA	NA	6.31 ± 2.3	NA
Baseline disease activity (CDAI score)	NA	NA	327.6 ± 72.7	355.3 ± 101.9	NA	322.9 ± 63.5
Prior TNFα antagonist use						
Yes	11 (73.3)	2 (66.7)	26 (72.2)	4 (100)	29 (52.7)	63 (61.8)
No	4 (26.7)	1 (33.3)	10 (27.8)	0 (0.0)	8 (14.5)	20 (19.6)
Missing	0 (0.0)	0 (0.0)	0 (0.0)	0 (0.0)	18 (32.7)	19 (18.6)
Any prior TNFα antagonist failure	11 (73.3)	2 (66.7)	25 (69.4)	4 (100)	44 (80.0)	77 (75.5)
Concomitant immunomodulator only	1 (6.7)	1 (33.3)	5 (13.9)	0 (0.0)	5 (9.1)	8 (7.8)
Concomitant corticosteroid only	5 (33.3)	1 (33.3)	18 (50.0)	1 (25.0)	23 (41.8)	33 (32.4)
Concomitant corticosteroid and immunomodulator	2 (13.3)	0 (0.0)	4 (11.1)	0 (0.0)	4 (7.3)	14 (13.7)
Prior immunomodulator						
Yes	13 (86.7)	3 (100)	29 (80.6)	4 (100)	15 (27.3)	23 (22.5)
No	2 (13.3)	0 (0.0)	7 (19.4)	0 (0.0)	22 (40.0)	60 (58.8)
Missing	0 (0.0)	0 (0.0)	0 (0.0)	0 (0.0)	18 (32.7)	19 (18.6)
Prior corticosteroid, *n* (%)						
Yes	14 (93.3)	3 (100)	36 (100)	4 (100)	24 (43.6)	46 (45.1)
No	1 (6.7)	0 (0.0)	0 (0.0)	0 (0.0)	13 (23.6)	37 (36.3)
Missing	0 (0.0)	0 (0.0)	0 (0.0)	0 (0.0)	18 (32.7)	19 (18.6)
Number of days of exposure to study drug	258.5 ± 118.0	181.4 ± 118.4	246.8 ± 112.4	203.0 ± 127.0	1,262 ± 836	1,100 ± 804
Time of surgery after most recent dose of vedolizumab						
Days, mean (SD)	38.4 ± 31.5	NA	25.8 ± 18.5	NA	42.1 ± 21.9	50.0 ± 40.4
Days, median (range)	23 (8–88)	NA	20 (1–92)	NA	39 (8–106)	40 (2–283)
1–30 days, *n* (%)	9 (60.0)	NA	25 (69.4)	NA	16 (29.1)	38 (37.3)
31–60 days, *n* (%)	1 (6.7)	NA	9 (25.0)	NA	31 (56.4)	32 (31.4)
>60 days, *n* (%)	5 (33.3)	NA	2 (5.6)	NA	8 (14.5)	32 (31.4)

Values presented as mean ± SD or *N* (%).

IBD, inflammatory bowel disease; UC, ulcerative colitis; CD, Crohn’s disease; LTS, long-term safety; CDAI, Crohn’s Disease Activity Index; TNFα, tumor necrosis factor alpha; NA, not applicable; SD, standard deviation.

In GEMINI 1, colectomy occurred in 2.4% of patients (15/620) in the vedolizumab group and in 2.0% (3/149) of the placebo group. In GEMINI 2, bowel surgery/resection was reported in 4.4% of patients receiving vedolizumab (36/814) and in 2.7% of patients receiving placebo (4/148).

At data cutoff (19 May 2016), the GEMINI LTS study included 894 patients with UC and 1,349 patients with CD. A total of 157 patients underwent IBD-related surgery, with 6.2% of patients with UC (55/894) and 3.0% of patients with CD (41/1349) having a colectomy procedure, and no patients with UC and 4.5% of patients with CD (61/1,349) having bowel surgery or resection. Vedolizumab exposure time in the GEMINI LTS study was longer than in the GEMINI 1 and GEMINI 2 trials, with patients with UC and CD receiving vedolizumab for a mean of 1,262 days and 1,100 days, respectively. In GEMINI LTS, the most recent vedolizumab infusion was 2–283 days (median, 40 days) before surgery. The median number of days between last treatment dose and surgery was 39 (8‒106) for patients with UC and 40 (2‒283) for patients with CD.

The proportion of patients requiring IBD-related surgery declined over time in the GEMINI LTS study, with the incidence of colectomy in patients with UC decreasing from 4.0% in the first year to 0% in the fifth year, and the incidence of bowel resection in patients with CD decreasing from 4.5% to 0.3% in the same period (Table 2).

**Table 2.** Incidence of IBD-related surgery over time in GEMINI LTS study only^a^

Parameter	Year 1	Year 2	Year 3	Year 4	Year 5	Year 6
UC patients	*n* = 845	*n* = 632	*n* = 512	*n* = 371	*n* = 238	*n* = 60
No. of patients with colectomy	34	7	5	3	0	0
Percentage (95% CI)	4.0 (2.7–5.3)	1.1 (0.3–1.9)	1.0 (0.3–2.3)	0.8 (0.2–2.3)	0 (0–1.5)	0 (0–6.0)
CD patients	*n* = 1,297	*n* = 912	*n* = 736	*n* = 560	*n* = 314	*n* = 67
No. of patients with bowel resection	58	18	6	3	1	0
Percentage (95% CI)	4.5 (3.3–5.6)	2.0 (1.1–2.9)	0.8 (0.2–1.5)	0.5 (0.1–1.6)	0.3 (0–1.8)	0 (0–5.4)

Data cutoff point: 21 May 2015.

a Patients with moderate-to-severe UC or CD who received at least one dose of vedolizumab in the GEMINI LTS study and had at least one post-baseline disease activity measurement.

IBD, inflammatory bowel disease; UC, ulcerative colitis; CD, Crohn’s disease; LTS, long-term safety; CI, confidence interval.

### Post-operative complications in the clinical trials

Four post-operative complications were reported in patients undergoing IBD-related surgery in both the vedolizumab and placebo groups (GEMINI 1: 2/15 [13.3%] and 1/3 [33.3%] patients, respectively; GEMINI 2: 1/36 [2.8%] and 0/4 patients, respectively; Table 3). Complications in vedolizumab-treated patients were wound infection (UC), serious adverse effect of sepsis (UC), and bacteremia (CD). A serious post-operative complication of sepsis was reported in one patient with UC receiving placebo.

**Table 3.** Post-operative complications for patients undergoing IBD-related surgery in the clinical studies

Complication	GEMINI 1: UC		GEMINI 2: CD		GEMINI LTS study: all vedolizumab-treated^a^		
	Vedolizumab (*n* = 15)	Placebo (*n* = 3)	Vedolizumab (*n* = 36)	Placebo (*n* = 4)	UC (*n* = 55)	CD (*n* = 102)	Total (*N* = 157)
Post-operative complications (all and subgroup of serious)							
Any post-operative complication, *n* (%)	2 (13.3)	1 (33.3)	1 (2.8)	0 (0.0)	2 (3.6)	9 (8.8)	11 (7.0)
Serious post-operative complication, *n* (%)	1 (6.7)^b^	1 (33.3)^b^	0 (0.0)	0 (0.0)	1 (1.8)	5 (4.9)	6 (3.8)^c^
All post-operative complications^d^							
Post-operative ileus, *n* (%)	0 (0.0)	0 (0.0)	0 (0.0)	0 (0.0)	0 (0.0)	4 (3.9)	4 (2.5)
Post-operative wound infection, *n* (%)	1 (6.7)	0 (0.0)	0 (0.0)	0 (0.0)	1 (1.8)	2 (2.0)	3 (1.9)
Abdominal sepsis, *n* (%)	0 (0.0)	0 (0.0)	0 (0.0)	0 (0.0)	1 (1.8)	1 (1.0)	2 (1.3)
Abdominal-wound dehiscence, *n* (%)	0 (0.0)	0 (0.0)	0 (0.0)	0 (0.0)	0 (0.0)	2 (2.0)	2 (1.3)
Bacteremia, *n* (%)	0 (0.0)	0 (0.0)	1 (2.8)	0 (0.0)	0 (0.0)	1 (1.0)	1 (0.6)
Sepsis, *n* (%)	1 (6.7)	1 (33.3)	0 (0.0)	0 (0.0)	0 (0.0)	1 (1.0)	1 (0.6)
Septic shock, *n* (%)	0 (0.0)	0 (0.0)	0 (0.0)	0 (0.0)	1 (1.8)	0 (0.0)	1 (0.6)
Wound dehiscence, *n* (%)	0 (0.0)	0 (0.0)	0 (0.0)	0 (0.0)	0 (0.0)	1 (1.0)	1 (0.6)

Data cutoff point for GEMINI LTS study: 19 May 2016.

a Patients with at least one adverse event within one level of the MedDRA-preferred term are counted only once in that level.

b One patient receiving vedolizumab (sepsis) and one patient receiving placebo (sepsis) in GEMINI 1.

c Abdominal sepsis, abdominal-wound dehiscence, sepsis, post-operative wound infection, post-operative ileus, and wound dehiscence.

d Grouped by MedDRA-preferred term.

IBD, inflammatory bowel diseases; LTS, long-term safety; UC, ulcerative colitis; CD, Crohn’s disease; MedDRA, *Medical Dictionary for Regulatory Activities*.

In the GEMINI LTS study, 11/157 (7.0%) vedolizumab-treated patients experienced 15 post-operative complications (Table 3). The most frequent complication was post-operative ileus, reported in four patients (2.5%). Overall, eight serious post-operative complications were reported in 6/157 patients (3.8%); these were abdominal sepsis (*n *=* *2; UC and CD), abdominal-wound dehiscence (*n *=* *2; CD), sepsis (*n *=* *1; CD), post-operative wound infection (*n *=* *1; CD), post-operative ileus (*n *=* *1; CD), and wound dehiscence (*n *=* *1; CD).

### Post-operative complications in the post-marketing setting

In the post-marketing setting, based on data covering approximately 46,978 patient-years of vedolizumab exposure, there were 20 reports of post-operative complications in 19 patients, with sepsis/bacterial sepsis being the most common (*n *=* *7; Table 4). At the time of reporting, 6 of the 19 patients had recovered or were recovering from their post-operative complication, 1 had not recovered, 6 had died, and the outcome of the post-operative complication was not reported in the remaining 6 patients. In the 13 patients with non-fatal post-operative complications, vedolizumab was continued by seven and discontinued by two individuals, and the outcome was not reported for four patients. Of 11 patients with available information (prior medication history was not reported in 8 patients), 9 had received TNFα antagonist treatment before receiving vedolizumab. Of these nine patients, seven received concomitant steroid therapy.

**Table 4.** Post-operative complications reported after surgery in the post-marketing setting

Complication^a^	Serious events (16 events)		Non-serious events (4 events)		Total events (20 events)	
Ulcerative colitis						
Prior TNFα antagonist use	Yes	No	Yes	No	Yes	No
Post-procedural complication^b^	1	1	0	0	1	1
Impaired healing	0	1	0	0	0	1
Post-procedural hemorrhage	0	0	1	0	1	0
Post-operative wound infection	0	1	0	0	0	1
Crohn’s disease						
Prior TNFα antagonist use	Yes	No	Yes	No	Yes	No
Sepsis	2	3	0	0	2	3
Post-procedural complication^b^	0	1	0	0	0	1
Impaired healing	1	0	1	0	2	0
Post-procedural hemorrhage	1	0	0	0	1	0
Septic shock^c^	0	1	0	0	0	1
Wound dehiscence	1	0	0	0	1	0
Wound infection	0	0	1	0	1	0
Other/unknown indications						
Prior TNFα antagonist use	Yes	No	Yes	No	Yes	No
Sepsis	0	1^d^	0	0	0	1
Post-procedural complication^b^	0	0	0	1^e^	0	1
Bacterial sepsis	0	1^f^	0	0	0	1

Data cutoff point: 19 May 2016.

a Grouped by MedDRA (*Medical Dictionary for Regulatory Activities*)-preferred term.

b Post-procedural complications (verbatim terms) included ‘post-operative leaky gut’, ‘blood clot or something else related to surgery’, and ‘2 days after the surgery, patient had blockage’.

c One patient had two complications (sepsis and septic shock).

d Off-label use (GVHD).

e Indication not reported.

f IBD not specified.

TNFα, tumor necrosis factor alpha; GVHD, graft-versus-host disease; IBD, inflammatory bowel disease.

### Deaths in the clinical trials and post-marketing setting

In the clinical trials, two deaths (one patient receiving vedolizumab and one patient receiving placebo) occurred <30 days after surgery (Table 5). A 75-year-old man with CD who was receiving placebo in GEMINI 2 died of bronchopneumonia, having previously undergone a loop ileostomy; this death was not considered to be related to the study drug (placebo) by the investigator. In the second case, a proctocolectomy, J-pouch, and ileostomy had been performed in a 49-year-old woman with UC receiving vedolizumab in the GEMINI LTS study. The cause of death for this patient was respiratory failure, which was not considered by the investigator to be related to the study drug.

**Table 5.** Deaths from post-operative complications in the clinical trials and post-marketing setting occurring <30 days after surgery

Patient details (sex, age, indication)	Study in which case was reported (treatment group)^a^	Cause of death	Concomitant and prior medications	Medical history	Surgical history
Clinical trials					
Male, 75, CD	GEMINI 2 (placebo)	Bronchopneumonia	Concomitant: oral mesalamine, paracetamol, terbutaline sulfate, formoterol fumarate dehydrate, aspirin, insulin, simvastatin Prior: NR	Type 2 diabetes mellitus, augmentin hypersensitivity, urinary incontinence, asthma, conjunctivitis, chronic obstructive pulmonary disease, bronchiectasis, cerebrovascular accident	Loop ileostomy
Female, 49, UC	GEMINI LTS study (vedolizumab)	Respiratory failure	Concomitant: hydrocortisone Prior: corticosteroids (NS), azathioprine, mesalazine, sulfasalazine	Autoimmune thyroiditis	Proctocolectomy, J-pouch, ileostomy
Post-marketing setting					
Female, 73, CD	NA	Multiorgan failure, sepsis, duodenal ulcer perforation	Concomitant: corticosteroids (NS) Prior: anti-TNFα (NS)	Diabetes mellitus, pituitary-dependent Cushing’s syndrome, hypertension, Parkinson’s disease, asthma, gastroesophageal reflux disease, hiatus hernia, anorectal disorder, diverticulitis	Full colectomy, fistula surgery
Male, 57, CD	NA	Sepsis; septic shock due to peritonitis with multiorgan failure against background of intestinal perforation; inflammatory conglomerate mass in the ileum; abscess in the area of the descendostomy	Concomitant: NR Prior: methotrexate	Bronchial carcinoma, osteoporosis, chronic obstructive pulmonary disease, tinnitus, hypoacusis, smoker, aortic arteriosclerosis, peliosis hepatitis, pancreatic steatosis, metronidazole intolerance, primary hypogonadism, proctectomy, chemotherapy, thoracotomy	Rectum and sigma extirpation with insertion of a terminal descendostomy
Female, UC (age NR)	NA	Thrombosis/unspecified post-operative complication^b^	NR	NR	Total abdominal colectomy
CD (details NR)	NA	Sepsis	NR	NR	Operation (type unknown)
CD (details NR)	NA	Sepsis	NR	NR	Operation (type unknown)
Unspecified IBD (details NR)	NA	Bacterial sepsis	NR	NR	NR

a All patients were receiving vedolizumab in the post-marketing setting.

b The patient died from a blood clot/something else related to surgery.

CD, Crohn’s disease; UC, ulcerative colitis; IBD, inflammatory bowel disease; LTS, long-term safety; NA, not applicable; NR, not reported; NS, not specified; TNFα, tumor necrosis factor alpha.

In the post-marketing setting, six deaths were reported. Causes of death were sepsis/septic shock (*n *=* *4), multiorgan failure in combination with sepsis and duodenal ulcer perforation (*n *=* *1), and thrombosis/unspecified post-operative complication (*n *=* *1; Table 5).

## Discussion

Patients with IBD often require surgical intervention during the course of their disease, and because many of these patients will have received or will be using immunosuppressive therapies around the time of surgery, it is important to understand the risk of each therapy to surgical outcomes. Evidence of the impact of vedolizumab on post-operative complications, derived from retrospective analyses of observational databases, is inconclusive, with some studies reporting that the risk of post-operative complications is increased with vedolizumab and others reporting no increased risk. Our analyses—which are the first to use data from prospective, randomized clinical trials of vedolizumab and from the post-marketing setting—indicate that rates of post-operative complications in vedolizumab-treated patients are low.

The incidence of IBD-related surgery was low in the GEMINI clinical trials, occurring in approximately twice as many patients with CD (*n *=* *142) as UC (*n *=* *73), consistently with published data [1]. There are limited reports on the rate of surgery over time with vedolizumab treatment. However, a large, multicenter consortium, involving 742 patients with IBD initiating vedolizumab in clinical practice, observed a significant decline in the proportion of patients undergoing surgery over time (*P *<* *0.05 for both UC and CD) [25]. Accordingly, the proportion of patients requiring IBD-related surgery declined over time in the GEMINI LTS study. However, this study was not designed to examine such trends. In addition, patients were withdrawn if they required major surgical intervention for treatment of UC or CD, required rescue medication, or experienced a treatment-related adverse effect leading to discontinuation. Patients were also withdrawn if the patient became pregnant or, in the opinion of the investigator or patient, was not benefiting from therapy [26, 27].

Post-operative complications after surgery for UC or CD can influence morbidity and mortality as well as length of hospital stay and cost of care [31, 32]. Although there are several publications of post-operative complication rates with vedolizumab treatment, all are based on data from retrospective database studies, and many describe a data set from a single institution. A systematic review identified five studies that included 307 vedolizumab-treated patients with IBD, 490 anti-TNFα-treated patients with IBD, and 535 patients with IBD who were not exposed to pre-operative biologic therapy, in total. Calculation of pooled risk ratios (RRs) showed that risks of post-operative infectious complications (RR 0.99; 95% confidence interval [CI], 0.37–2.65) and overall post-operative complications (RR 1.00; 95% CI, 0.46–2.15) were not significantly different between vedolizumab-treated patients and those who received no pre-operative biologic therapy. Furthermore, the risks of post-operative infectious complications (RR 0.99; 95% CI, 0.34–2.90) and overall post-operative complications (RR 0.92; 95% CI, 0.44–1.92) were not significantly different between vedolizumab-treated and anti-TNFα-treated patients [22]. In vedolizumab clinical trials (GEMINI 1, GEMINI 2, and the GEMINI LTS study), ileus and wound infection were the most frequently reported post-operative complications, each being observed in four vedolizumab-treated patients. No cases of anastomotic leak were reported in GEMINI 1 or GEMINI 2. In the post-marketing setting, the most common post-operative complication was sepsis/bacterial sepsis (*n *=* *7). Although analysis of the effect of corticosteroid use on wound infection rates was beyond the scope of this study, data on corticosteroid use in patients with concomitant colectomy or bowel surgeries are provided in Supplementary Table 2.

Hypothetically, the mode of action of vedolizumab has been thought to result in reduced availability of leukocytes in the gut, and vedolizumab treatment might affect the healing process for stomas and intestinal anastomoses [4]. However, vedolizumab acts by blocking the interaction of α_4_β_7_ with mucosal addressin cell adhesion molecule-1, thereby selectively inhibiting the migration of memory T lymphocytes across the endothelium into inflamed gastrointestinal tissue and sparing other leukocytes involved in wound healing [33, 34]. For example, a mechanistic study in non-human primates demonstrated that vedolizumab affects approximately 1% of total peripheral blood leukocytes [35]. This effect on a small subset of memory T lymphocytes is also evidenced in human clinical trials in which no increase was seen in total peripheral blood neutrophils, basophils, eosinophils, B-helper and cytotoxic T lymphocytes, total memory helper T lymphocytes, monocytes, or natural killer cells [36]. The similar frequency of post-operative complications between patients exposed to vedolizumab and placebo in GEMINI 1 and GEMINI 2 is consistent with a mode of action that does not affect all gut leukocytes.

Several factors might influence the risk of post-operative complications in patients receiving vedolizumab in our data sources. In particular, several retrospective observational studies concluded that pre-operative use of TNFα antagonists may modestly increase the risk of post-operative complications in patients with IBD [7–11]. To date, no prospective study evaluating pre-operative biologic therapy and post-operative complications has been reported, but results from a study to determine the risk factors for post-operative infection in IBD (the PUCCINI study) are eagerly awaited [37]. In both the clinical trials and post-marketing setting, the frequency of complications in patients with prior TNFα antagonist exposure was not significantly higher than in those without such exposure. Owing to the design and small sample of our analysis, adjustments for confounding variables and previous or concurrent use of immunomodulators and corticosteroids were not possible.

Post-marketing data offer the opportunity to evaluate LTS in real-world clinical practice [38]. Limitations of post-marketing reporting can result in difficulty establishing a causal relationship between treatment exposure and occurrence of adverse effects. These limitations include incomplete reporting of data and difficulty accessing individual patient-level data, such as comorbidity, the temporal relationship between exposure to prior treatments and adverse effect onset, medical history, and comedication. There is also an increased likelihood of reporting serious adverse effects, relative to less serious adverse effects, because the reporting of adverse effects is voluntary. Limitations specific to this analysis include lack of detail regarding the timing between surgery and onset of the complication, information on comorbidities, severity of post-operative complications, and operation specifics. For 4/6 patients (66.7%) who had a fatal post-operative complication, no information on past medical history, comorbidities, or comedications was reported. Limitations specific to the GEMINI trials include lack of data on disease severity, drug levels, nutritional status, and albuminemia at the time of surgery. The GEMINI trials did not systematically collect detailed information on surgery type and procedure stages.

In conclusion, in the clinical trials, complications of colectomy and bowel surgery/resection appeared to be infrequent, with minimal difference between vedolizumab and placebo. The frequency of post-operative complications in the post-marketing setting appears low. Active pharmacovigilance, ongoing observational studies (including a prospective international cohort study and a post-authorization safety study, comparing vedolizumab with other biologic treatments) and the GEMINI LTS study should continue to inform treatment decisions for patients with IBD requiring bowel surgery.

## Authors’ contributions

B.S., A.B., K.L., M.S., and F.B. conceived of and designed the project. A.B. and F.B. collected the data. B.S., A.B., K.L., M.S., and F.B. analysed and interpreted the data. B.S., A.B., K.L., M.S., and F.B. drafted the manuscript. All authors read and approved the final manuscript.

## Funding

This work was supported by Takeda Pharmaceutical Company Ltd.

## Supplementary Material

goz034_Supplementary_Data
