# Supplementary material for: Vedolizumab use in patients with inflammatory bowel diseases undergoing surgery: clinical trials and post-marketing experience
Source: Gastroenterol Rep (Oxf). 2019 Aug 21;7(5):322–30. doi: 10.1093/gastro/goz034 (PMC6821312; doi:10.1093/gastro/goz034)

**Supplementary File 1 - Supplementary methods**

Inclusion and exclusion criteria for GEMINI 1 and GEMINI 2 trials

Patients were excluded if they had an extensive colonic resection, subtotal or total colectomy, ileostomy, colostomy, or known fixed symptomatic stenosis of the intestine, or if they had undergone any surgical procedure requiring general anesthesia <30 days before enrollment or anticipated major surgery during the study period.

GEMINI long-term safety study

Rollover patients were excluded if they currently required, were expected to require, or had previously undergone (during the prior vedolizumab trial) major surgery for control of inflammatory bowel disease (IBD). Patients who had undergone or may have needed minor surgical procedures (non-IBD-related and minor IBD-related procedures, e.g. fistulotomy) were included. De novo patients were excluded if they had any surgical procedure requiring general anesthesia <30 days before enrollment, or if they had any expected requirement for major surgery during the study period.

GEMINI long-term safety (LTS) study data are based on an interim analysis of data collected up to May 19, 2016, and data on all patients who received at least one vedolizumab dose in the study were included.

**Supplementary File 2 - Appendix**

Patient records were searched for using the following *Medical Dictionary for Regulatory Activities* (MedDRA) preferred terms. MedDRA version 14.0 was used for coding adverse events in the clinical studies, and version 19.0 was used for the postmarketing reports. The same search terms were used for the clinical trial data and the postmarketing data.

**Sepsis, bacteremia, viremia, and fungemia not elsewhere classified group MedDRA preferred terms**

Abdominal sepsis

Acinetobacter bacteremia

Amniotic infection syndrome of Blane

Bacteremia

Bacterial toxemia

Bacterial translocation

Bacteroides bacteremia

Cerebral septic infarct

Device related sepsis

Endotoxemia

Endotoxic shock

Fungemia

Intestinal sepsis

Neutropenic sepsis

Pelvic sepsis

Postprocedural sepsis

Postpartum sepsis

Pulmonary sepsis

Sepsis

Sepsis neonatal

Sepsis syndrome

Septic embolus

Septic encephalopathy

Septic necrosis

Septic phlebitis

Septic rash

Septic shock

Septic vasculitis

Thrombophlebitis septic

Urosepsis

Viremia

Wound sepsis

**Wound-healing MedDRA preferred terms**

Abdominal wound dehiscence

Impaired healing

Postoperative abscess

Postoperative wound infection

Postoperative wound complication

Postoperative ileus

Postprocedural complication

Postprocedural discharge

Postprocedural hemorrhage

Postprocedural infection

Postprocedural sepsis

Wound

Wound abscess

Wound complication

Wound decomposition

Wound dehiscence

Wound infection

Wound secretion

Wound sepsis

**Supplementary Table 1.** Baseline characteristics in GEMINI 1, GEMINI 2, and GEMINI LTS

| Parameter | GEMINI 1: UC | | GEMINI 2: CD | | Pooled GEMINI 1 and 2 | | GEMINI LTS | | |
| --- | --- | --- | --- | --- | --- | --- | --- | --- | --- |
|  | Vedolizumab (*n* = 620) | Placebo  (*n* = 149) | Vedolizumab (*n* = 814) | Placebo  (*n* = 148) | Vedolizumab (*n* = 1434) | Placebo  (*n* = 297) | UC  (*n* = 894) | CD  (*n* = 1349) | Total  (*N* = 2243) |
| Age, years | 40.1 ± 13.1 | 41.2 ± 12.5 | 35.5 ± 11.9 | 38.6 ± 13.2 | 37.5 ± 12.6 | 39.9 ± 12.9 | 41.2 ± 13.6 | 37.8 ± 12.7 | 39.1 ± 13.2 |
| Female | 256 (41.3) | 57 (38.3) | 435 (53.4) | 79 (53.4) | 691 (48.2) | 136 (45.8) | 372 (41.6) | 743 (55.1) | 1115 (49.7) |
| White | 518 (83.5) | 115 (77.2) | 731 (89.8) | 124 (83.8) | 1249 (87.1) | 239 (80.5) | 762 (85.2) | 1219 (90.4) | 1981 (88.3) |
| Body weight, kg | 73.4 ± 18.3 | 72.4 ± 17.7 | 70.1 ± 19.8 | 68.7 ± 18.9 | 71.5 ± 19.2 | 70.6 ± 18.3 | 75.2 ± 18.1 | 71.8 ± 19.3 | 73.2 ± 18.9 |
| Current smoker^a^ | 36 (5.8) | 11 (7.4) | 216 (26.5) | 34 (23.0) | 252 (17.6) | 45 (15.2) | 43 (5.0)^b^ | 363 (27.1)^b^ | 406 (18.4)^b^ |
| Former smoker | 204 (32.9) | 50 (33.6) | 190 (23.3) | 29 (19.6) | 394 (27.5) | 79 (26.6) | 272 (31.4)^b^ | 319 (23.8)^b^ | 591 (26.8)^b^ |
| Duration of disease, years^c^ | 6.7 ± 6.0 | 7.1 ± 7.3 | 9.1 ± 7.5 | 8.2 ± 7.8 | 8.0 ± 7.0 | 7.7 ± 7.5 | 8.0 ± 6.9 | 10.1 ± 8.3 | 9.3 ± 7.9 |
| Disease duration ≥7 years | 214 (34.5) | 53 (35.6) | 419 (51.5) | 64 (43.2) | 633 (44.1) | 117 (39.4) | 372 (41.6) | 755 (56.0) | 1127 (50.2) |
| Baseline disease activity (complete Mayo Score) | 8.6 ± 1.8 | 8.6 ± 1.7 | − | − | − | − | − | − | − |
| Baseline disease activity (partial Mayo Score)^a^ | 6.0 ± 1.6 | 6.1 ± 1.5 | − | − | − | − | 5.8 ± 1.8 | − | − |
| Baseline disease activity (HBI score) ^a^ | – | – | 11.2 ± 3.8 | 10.9 ± 3.7 | − | – | − | 10.9 ± 3.6 | 10.9 ± 3.6 |
| Baseline disease activity (CDAI score) ^d^ | – | – | 323.1 ± 68.5 | 324.6 ± 78.1 | – | – | − | 314.0 ± 63.2^e^ | 314.0 ± 63.2 |
| Baseline disease activity score^f^ | − | − | − | − | 5.8 ± 1.7 | 5.8 ± 1.7 | − | − | − |
| Fistulizing disease at baseline | – | – | 297 (36.5) | 56 (37.8) | – | – | – | 500 (37.1) | 500 (37.1) |
| Prior TNFα antagonist use | 311 (50.2) | 73 (49.0) | 535 (65.7) | 72 (48.6) | 846 (59.0) | 145 (48.8) | 415 (46.4) | 898 (66.6) | 1313 (58.5) |
| Any prior TNFα antagonist failure | 266 (42.9) | 63 (42.3) | 497 (61.1) | 70 (47.3) | 763 (53.2) | 133 (44.8) | 380 (42.5)^g^ | 848 (62.9)^g^ | 1228 (54.7)^g^ |
| Concomitant immunomodulators | 213 (34.4) | 44 (29.5) | 270 (33.2) | 51 (34.5) | 483 (33.7) | 95 (32.0) | 237 (26.5) | 381 (28.2) | 618 (27.6) |
| Concomitant corticosteroids | 325 (52.4) | 84 (56.4) | 417 (51.2) | 71 (48.0) | 742 (51.7) | 155 (52.2) | 452 (50.6) | 681 (50.5) | 1133 (50.5) |
| Concomitant narcotics | 123 (19.8) | 25 (16.8) | 276 (33.9) | 39 (26.4) | 399 (27.8) | 64 (21.5) | 247 (27.6) | 530 (39.3) | 777 (34.6) |

Values presented as mean ± standard deviation or N (%).

^a^ For GEMINI LTS, collected at baseline of previous vedolizumab study for rollover patients and at baseline of the GEMINI LTS study for de novo patients.

^b^ Patients with data: UC, *n* = 865; CD, *n* = 1341; total, *N* = 2206. Excludes rollover phase 2 extension-study patients; relevant data for phase 2 extension-study rollover patients were not collected or were not incorporated into the GEMINI LTS database.

^c^ For GEMINI LTS data, disease duration defined as (1 + first dose date in the GEMINI LTS study – diagnosis date)/365.25.

^d^ For GEMINI LTS, as collected at baseline of previous vedolizumab study for rollover patients; CDAI was not collected for de novo patients.

^e^ Patients with data: CD *n* = 1109. Excludes rollover phase 2 extension-study patients as well as de novo patients.

^f^ Baseline disease activity scores based on partial Mayo Score for GEMINI 1 (UC), HBI score for GEMINI 2 (CD), and common index for pooled GEMINI 1 and 2. Common index ranged from 0 to 9 to allow the combination of baseline partial Mayo and HBI scores in the pooled analysis.

^g^ Patients with data: UC, *n* = 822; CD, *n* = 1309; total, *N* = 2131.

UC, ulcerative colitis; CD, Crohn’s disease; LTS, long-term safety; CDAI, Crohn’s Disease Activity Index; HBI, Harvey–Bradshaw Index; TNFα, tumor necrosis factor alpha

**Supplementary Table 2.** Corticosteroid dose (mg/day) for patients with concomitant colectomy or bowel surgeries.

| Parameter | GEMINI 1: UC | | GEMINI 2: CD | | Pooled GEMINI 1 and 2 | | GEMINI LTS | | |
| --- | --- | --- | --- | --- | --- | --- | --- | --- | --- |
|  | Vedolizumab  (*n* = 15) | Placebo  (*n* = 3) | Vedolizumab  (*n* = 36) | Placebo  (*n* = 4) | Vedolizumab  (*n* = 51) | Placebo  (*n* = 7) | UC | CD | Total |
| Any concomitant corticosteroid | *n* = 7 | *n* = 1 | *n* = 22 | *n* = 1 | *n* = 29 | *n* = 2 | *n* = 27 | *n* = 47 | *n* = 74 |
| ***Corticosteroid, mg/day (day range)*** | | | | | | | | | |
| Baseline (1–1) | *n* = 0 | *n* = 1 | *n* = 0 | *n* = 0 | *n* = 0 | *n* = 1 | *n* = 0 | *n* = 8 | *n* = 8 |
| Mean ± SD | – | 20.0 | – | – | 0 | 20.0 | – | 23.8 ± 10.6 | 23.8 ± 10.6 |
| Median | – | 20.0 | – | – | 0 | 20.0 | – | 25.0 | 25.0 |
| Min, max | – | 20, 20 | – | – | 0 | 20, 20 | – | 10, 40 | 10, 40 |
| Week 26 (169–196) | *n* = 2 | *n* = 0 | *n* = 4 | *n* = 0 | *n* = 6 | *n* = 0 | *n* = 7 | *n* = 12 | *n* = 19 |
| Mean ± SD | 6.0 ± 5.5 | – | 12.5 ± 9.3 | – | 10.4 ± 8.3 | – | 11.2 ± 8.5 | 12.0 ± 14.6 | 11.7 ± 12.5 |
| Median | 6.0 | – | 11.4 | – | 8.2 | – | 8.8 | 6.3 | 7.0 |
| Min, max | 2, 10 | – | 4, 24 | – | 2, 24 | – | 1, 21 | 0, 53 | 0, 53 |
| Week 52 (359–388) | *n* = 1 | *n* = 0 | *n* = 1 | *n* = 0 | *n* = 2 | *n* = 0 | *n* = 2 | *n* = 3 | *n* = 5 |
| Mean (SD) | 5.0 | – | 16.7 | – | 10.8 ± 8.3 | – | 7.3 ± 8.6 | 11.5 ± 10.0 | 9.8 ± 8.6 |
| Median | 5.0 | – | 16.7 | – | 10.8 | – | 7.3 | 14.6 | 13.3 |
| Min, max | 5, 5 | – | 17, 17 | – | 5, 17 | – | 1, 13 | 0, 20 | 0, 20 |
| Just before surgery | *n* = 6 | *n* = 1 | *n* = 19 | *n* = 1 | *n* = 25 | *n* = 2 | *n* = 26 | *n* = 41 | *n* = 67 |
| Mean (SD) | 17.9 ± 10.1 | 20.0 | 25.9 ± 22.7 | 25.0 | 24.0 ± 20.5 | 22.5 ± 3.5 | 22.3 ± 30.9 | 13.7 ± 14.8 | 17.1 ± 22.6 |
| Median | 20.0 | 20.0 | 20.0 | 25.0 | 20.0 | 22.5 | 12.5 | 5.0 | 7.5 |
| Min, max | 3, 30 | 20, 20 | 5, 100 | 25, 25 | 3, 100 | 20, 25 | 1, 150 | 0, 75 | 0, 150 |

UC, ulcerative colitis; CD, Crohn’s disease; LTS, long-term safety; SD, standard deviation.

**Supplementary Figure 1.** Clinical data sources.

UC, ulcerative colitis, CD, Crohn’s disease; LTS, long-term safety; RCT, randomized controlled trial.

^a^ Patients who received vedolizumab or placebo during the induction and maintenance phases (excluding those who received vedolizumab in the induction phase but were randomized to placebo in the maintenance phase).


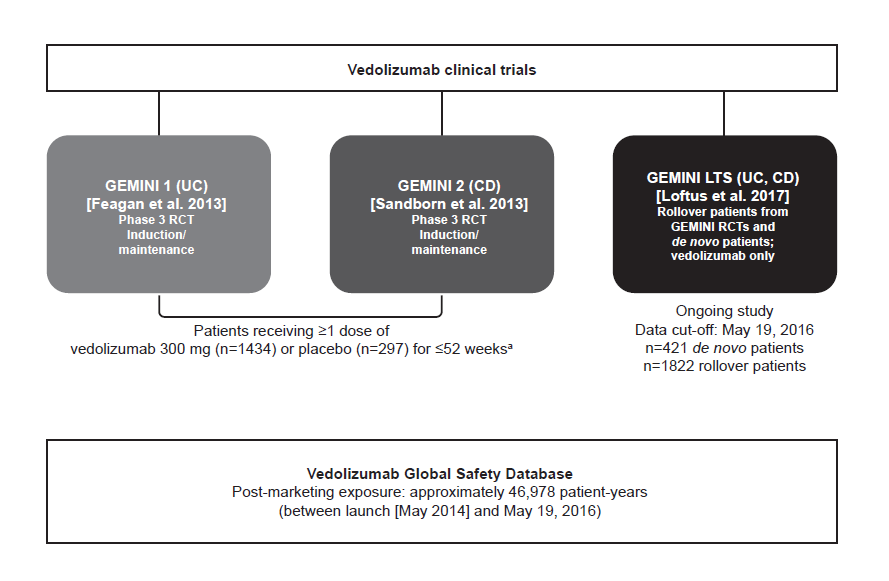

Supplement: goz034_Supplementary_Data [file goz034_supplementary_data.docx]
